# Supplementary material for: Mechanisms governing the pioneering and redistribution capabilities of the non-classical pioneer PU.1
Source: Nat Commun. 2020 Jan 21;11:402. doi: 10.1038/s41467-019-13960-2 (PMC6972792; doi:10.1038/s41467-019-13960-2)
Supplement: Supplementary file 7 — Source data [file 41467_2019_13960_MOESM7_ESM.zip › Source_Data/FigureS2/FigureS2B_MotifScanOutput/KmeansCluster14/homerResults/motif1.similar.html]

motif1

## Information for motif1

C
T
G
A
T
C
A
G
T
G
A
C
C
T
A
G
A
C
T
G
C
G
T
A
C
G
T
A
T
C
A
G
A
G
C
T
A
T
C
G
T
A
C
G
C
T
G
A
  
Reverse Opposite:  

G
A
C
T
A
T
G
C
T
A
G
C
T
C
G
A
A
G
T
C
C
G
A
T
A
C
G
T
A
G
T
C
A
G
T
C
A
C
T
G
A
G
T
C
G
A
C
T
  

|  |  |
| --- | --- |
| p-value: | 1e-153 |
| log p-value: | -3.538e+02 |
| Information Content per bp: | 1.634 |
| Number of Target Sequences with motif | 331.0 |
| Percentage of Target Sequences with motif | 64.90% |
| Number of Background Sequences with motif | 6141.1 |
| Percentage of Background Sequences with motif | 13.56% |
| Average Position of motif in Targets | 100.1 +/- 41.5bp |
| Average Position of motif in Background | 99.1 +/- 69.9bp |
| Strand Bias (log2 ratio + to - strand density) | -0.0 |
| Multiplicity (# of sites on avg that occur together) | 1.33 |
| Motif File: | file (matrix) reverse opposite |

### Similar de novo motifs found

|  |  |  |  |  |  |  |  |
| --- | --- | --- | --- | --- | --- | --- | --- |
| Rank | Match Score | Redundant Motif | P-value | log P-value | % of Targets | % of Background | Motif file |
| 1 | 0.968 | A T C G T G A C T C G A A T G C G C A T A C G T A G T C A G T C A C G T A T G C G A C T | 1e-149 | -343.888233 | 81.18% | 25.63% | motif file (matrix) |
| 2 | 0.980 | T A G C C T G A A T G C C G A T A C G T A G T C A G T C A C T G A G T C | 1e-145 | -335.344184 | 73.73% | 20.24% | motif file (matrix) |
| 3 | 0.964 | G A T C A T G C T G A C C T G A A G T C G C A T A C G T A T G C G A T C A C G T A G T C G A C T A G C T | 1e-140 | -324.294864 | 58.04% | 11.17% | motif file (matrix) |
| 4 | 0.965 | A T G C T A G C T G C A A G T C C G A T A C G T A G T C A G T C A C G T A T G C | 1e-137 | -316.925731 | 54.12% | 9.54% | motif file (matrix) |
| 5 | 0.957 | T G A C C G T A A T G C C G A T A C G T A G T C A G T C A C T G | 1e-130 | -300.535846 | 68.43% | 18.61% | motif file (matrix) |
| 6 | 0.930 | C T G A T A C G T G C A A C T G A C T G C G T A C G T A C T A G A G C T A C T G T C A G A T C G A C T G G C A T | 1e-128 | -295.613000 | 53.33% | 10.03% | motif file (matrix) |
| 7 | 0.945 | A C T G A C T G C G T A C G T A A C T G A G C T A T C G | 1e-107 | -247.558089 | 65.49% | 20.01% | motif file (matrix) |
| 8 | 0.875 | A C G T T C G A T A G C A G C T A G C T A G T C A G T C A C T G T A G C | 1e-91 | -211.422190 | 65.29% | 22.61% | motif file (matrix) |
| 9 | 0.831 | C T G A T A C G G T A C A T C G C A T G C G T A G T C A | 1e-80 | -186.231867 | 76.47% | 34.73% | motif file (matrix) |
| 10 | 0.780 | A G C T C G A T G T A C G T A C A C T G G T A C G A T C A G T C G C A T | 1e-74 | -172.238978 | 56.27% | 19.21% | motif file (matrix) |
| 11 | 0.781 | C G A T G T C A C A T G A T C G C G T A T G C A A T C G A C G T T C A G G A T C | 1e-55 | -128.332062 | 36.27% | 9.92% | motif file (matrix) |
| 12 | 0.752 | A C T G A C T G A G C T A G C T G T A C A G T C A C T G A G T C A G T C A G T C | 1e-50 | -115.523665 | 29.80% | 7.28% | motif file (matrix) |
| 13 | 0.795 | A T C G C A T G G C T A C T A G T A C G T C G A T C G A C A T G C A G T T A C G C A T G | 1e-40 | -94.082996 | 40.98% | 15.78% | motif file (matrix) |
| 14 | 0.830 | A T C G G T A C C T A G A C T G C G T A C G T A T A C G T A G C | 1e-38 | -88.073307 | 40.59% | 16.15% | motif file (matrix) |
| 15 | 0.774 | A T C G T A C G T C G A T C A G T C A G T C G A T G C A A T C G C A G T T C A G G C T A | 1e-28 | -65.566363 | 50.98% | 27.36% | motif file (matrix) |
| 16 | 0.772 | A C G T C T A G A T G C G C A T A G T C A C G T A C G T A G T C G T A C A C T G | 1e-18 | -42.261027 | 10.00% | 2.13% | motif file (matrix) |
| 17 | 0.756 | A T C G G T C A C T A G T C A G T C G A T C G A C T A G C A G T A C T G T A C G A C G T | 1e-18 | -41.897228 | 10.78% | 2.50% | motif file (matrix) |
| 18 | 0.814 | A C T G A G T C C G T A A G T C A C G T A C G T A G T C A G T C A C G T A G T C A G T C | 1e-13 | -32.235790 | 6.47% | 1.16% | motif file (matrix) |
